# On the Dynamism of Paintings Through the Distribution of Edge Directions

**DOI:** 10.3390/jimaging10110276

**Published:** 2024-11-01

**Authors:** Adrien Deliege, Maria Giulia Dondero, Enzo D’Armenio

**Affiliations:** 1 Department of Romance Languages and Literatures, Faculty of Philosophy and Letters, University of Liège, 4000 Liège, Belgium; mariagiulia.dondero@uliege.be (M.G.D.); enzo.darmenio@uliege.be (E.D.); 2 F.R.S.-FNRS, Rue d’Egmont 5, 1000 Bruxelles, Belgium

**Keywords:** digital humanities, edge detection, computer vision, art analysis

## Abstract

The digitization of artworks has recently offered new computational perspectives on the study of art history. While much of the focus has been on classifying styles or identifying objects, the analysis of more abstract concepts, such as the perception of motion or dynamism in still images, remains largely unexplored. Semioticians and artists have long explored the representation of dynamism in still images, but they often did so through theoretical frameworks or visual techniques, without a quantitative approach to measuring it. This paper proposes a method for computing and comparing the dynamism of paintings through edge detection. Our approach is based on the idea that the dynamism of a painting can be quantified by analyzing the edges in the image, whose distribution can be used to identify patterns and trends across artists and movements. We demonstrate the applicability of our method in three key areas: studying the temporal evolution of dynamism across different artistic styles, as well as within the works of a single artist (Wassily Kandinsky), visualizing and clustering a large database of abstract paintings through PixPlot, and retrieving similarly dynamic images. We show that the dynamism of a painting can be effectively quantified and visualized using edge detection techniques, providing new insights into the study of visual culture.

## 1. Introduction

The digitization of artworks has progressively enabled large-scale computer vision analyses, opening new avenues for the study of art history and visual culture [1,2]. Early techniques focused on basic analyses, such as color and saturation studies [1,2,3]. In recent years, modern “artificial intelligence” (AI) techniques have expanded the scope of these analyses, allowing for more complex tasks like pose estimation [4,5,6,7,8,9,10,11], including a focus on a specific part of the body, such as face [12], eyes [9], or hands [13], as well as tasks like classification [8,14,15,16], retrieval [6,17,18,19,20], visual question answering [21,22], and detection [7,23,24,25].

Despite these advances, some modalities remain challenging to analyze for AI systems due to the difficulty in annotating and training on specific concepts, such as motion versus stillness and character recognition [26]. In this work, we explore one of these challenging modalities: the “dynamism” in images, that is, the perception of some kind of motion in a technically still artwork.

This topic has also been neglected in the history of image semiotics and art history [27], which are the birthplaces of the present research question. In these fields, plastic arts such as painting or sculpture are considered as arts of space only, decoupled from temporality, needed to convey a sense of motion and associated with arts such as poetry and music [28]. This Kantian distinction between the arts of space and the arts of time is played out between a spatial syntax constituted by magnitudes of extents and a temporal syntax constituted by intervals of duration. The former concerns the juxtaposition of parts in space, and the latter, the organization of successive events. Only rare semiotic theories [27,29,30,31] have made it possible to consider paintings as instruments for simulating temporality (past, present, future) and movement (more or less durative, iterative) altogether, by considering the plane of expression of images rather than the plane of content and the figurative and narrative aspect of the images [29,31].

In particular, authors in [31] first addressed the problem of narrative construction in single-scene still images. Before focusing on their content, they advised to clarify the problem from the point of view of expression: how can we understand and explain the “motion” effect of meaning in still images? In other words, “how can an iconic artwork that is in principle static convey a sense of duration?” [31]. This question underlies another: how can we account for the fact that a fixed two-dimensional support can construct temporal effects of intersection between two or more processes, as well as effects of succession? How can we go beyond the materiality of the image’s static support to grasp the significance of the plastic dynamics of each work, even when the paintings in question are portraits that seem to be utterly static? [32,33]. Authors in [31] approach these questions by theorizing that objects, figures, in paintings are usually linked to a dynamic process, either as a function or as a property: a ball implies rolling, a knife the act of cutting. They also suggest that some features are interpreted as the product of a dynamic process occurring during the production of the artwork. For example, the blurred appearance of cathedrals can be attributed either to a moving viewer or to the painting motion itself, but in no case to a movement of the object, as a cathedral cannot move. This second characteristic of image dynamics is important because it mobilizes the movement of the hand of the artist and the viewer’s perception of it as a movement, at the very least a vibratory movement. This means that the arts can transgress their primary essence by comprising spatio-temporal aspects that go beyond the aforementioned classic dichotomy.

Finally, in parallel to academics, artists themselves attempted to use or even theorize painting as a way of representing speed and motion. For instance, Sassetta’s depiction of successive states of actions in early Renaissance painting shows different stages of a narrative within a single frame in *The Meeting of St. Anthony and St. Paul* (ca. 1440), where time is expressed through successive yet static moments, creating a spatial narrative like an image split in multiple frames rather than the illusion of continuous motion. In contrast, much later, Marey’s chronophotography sought to represent motion through the trail of an object’s movement, creating a visual breakdown of movement over time where the memory of the movement is imprinted on the physical support. Then, at the beginning of the 20th century, Italian futurists such as Marinetti, Boccioni, Balla, and others developed an artistic style that glorifies war, capturing movement and energy as a time-lapse of repeating patterns and even published manifesti on that topic [34]. Concurrently, Kandinsky presented his revolutionary approach to painting by exposing in a seminal book the details of the inner dynamics of painting, focusing the discussion on the basic elements that are points and lines and their relation to the material plane serving as a receptacle for the artwork [35]. In the same period, Klee introduced in an essay his theory of “taking a line for a walk”, emphasizing the dynamic interaction of lines and shapes to convey movement and narrative within abstract compositions, further exploring the temporal dimension of visual art [36]. Eventually, let us not forget Pollock, whose drip painting technique would take this exploration of motion further, turning the act of painting itself into a dynamic performance, with the rhythm and energy of the artist’s movements captured directly on the canvas [37]. We naturally analyzed some works of these artists in the present paper.

**Figure 1:**
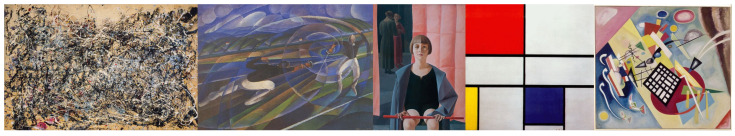
**How to visualize and quantify the dynamism of these paintings?** Human observers can “feel” that some of these images are more “dynamic” than others and can sometimes “see” dominant directions emerging from these paintings. In this paper, we propose an edge detection-based method to compute tangible characteristics that model these feelings and allow further large-scale analyses.

Dynamism can be interpreted and manifested in various ways, including motion blur, straight lines, curves, human gaze, effects like wind and waves, repetition, etc. [38,39]. Understanding and quantifying dynamism is important for art historians, as it provides insights into the emotional and expressive qualities of artworks [38,40]. Large-scale computer-assisted analyses can shed light on the temporal evolution of dynamism in art and help identify patterns and trends across artists and movements [26]. In this study, we focus on the dynamism induced by edges in paintings. We hypothesize that the main edges in a painting contribute to the perceived dynamism, with horizontal and vertical lines conveying a sense of calmness and stability, while diagonals are more dynamic [38]. By employing classical computer vision techniques, we aim to quantify and analyze this intuitive perception, as teased in Figure 1. Edges, fundamental in defining shapes and guiding visual attention, play a significant role in the interpretation of artworks [38]. They not only delineate objects but also contribute to the overall dynamism and emotional resonance of a composition. They define shapes, create depth, and guide the viewer’s gaze [38]. They can convey a sense of movement, tension, and emotion, influencing the overall dynamism of a painting [41]. Analyzing the dynamism of edges in paintings might thus, in future works, provide insights into the evolution of artistic styles and movements [26] and help researchers identify elements that reflect the cultural and social contexts in which the artworks were created.

From a technical point of view, we compute edges, in our case derived from Sobel filters [42], and aggregate them into histograms to characterize the dynamism in paintings. This process is one of the conventional approaches of a larger field, namely content-based image retrieval, where local and/or global image descriptors are computed and further used to characterize images and find similar images. Various such descriptors have been developed over the years, such as global image structure tensor (GIST) for, e.g., scene categorization [43] and web-scale image search [44], histograms of oriented gradients (HOGs) for, e.g., human detection [45] and sketch-based image retrieval [46,47,48], further complemented with Harris corners in [49], multi-scale oriented patches (MOPS) for, e.g., panoramic image stitching [50], bag-of-features (BoF) for, e.g., object matching in videos [51], local binary patterns (LBPs) for, e.g., texture analysis [52], to cite only a few prevalent ones, some of which also use Sobel filters [45,47,53]. While the use of such techniques is widespread in the general computer vision literature, few works seem to apply them in the artwork analysis domain. In the most notable works, HOGs and LBPs were used for painting classification into common art movements [53] and, along with GIST, for the evaluation of the aesthetic of artworks [54]. More distantly, [55] develops heuristics to extract brushstrokes, but the study is limited to a few Van Gogh paintings, as in the pioneering work [56] on brushstrokes analysis for artist identification. Many other computer vision methods have been used for tackling various other problems in arts, as reviewed in [57,58], but the study of the perceived paintings’ dynamism has been so far mostly neglected by the community. This is where the present paper is located within the research landscape.

Importantly, this paper is oriented more towards art historians than computer scientists, in the sense that we seek to study a research question (the dynamism of paintings) stemming from the former by showing what is possible with a method developed by the latter. This justifies our use of ad hoc yet relatively common image processing techniques, which we believe sufficient to yield first insightful results and pave the way for future more elaborated studies. In addition, it also explains why we focus on one technique rather than providing a comparison of various methods such as those listed above: the focus is to raise awareness by scholars of the art analysis community, i.e., to show what kind of processing can be made (computing, visualizing, aggregating, comparing edges and histograms) and what kind of domain-specific results can be derived (image comparisons, single artist examination, retrieval) rather than submerging the non computer vision experts with numerous comparisons and possibly insignificant differences between multiple methods. In addition, the absence of ground truth for any of these tasks for assessing quantitatively the differences between them would only yield subjective evaluations anyway. Consequently, when going through this paper, the interested reader should keep in mind that, from a technical point of view, many variants could be implemented, but the important point is to grasp the core concepts and the possibilities offered by such tools in the field of artwork analysis.

**Contributions.** We present a method for computing and comparing the dynamism in paintings through edge detection, accompanied by an open-source code repository and testing platform. In addition, we demonstrate the applicability of our method in three main ways: (1) we show how some different artistic styles can be distinguished and how the production of an artist (exemplified for Kandinsky) evolves through time from a “dynamism” perspective, (2) we show how to visualize hundreds of images and cluster them by dynamic similarity through the PixPlot software version 0.0.1.1, and (3) we retrieve similarly dynamic images in a corpus with respect to a query image. By providing a quantitative and scalable approach to analyzing dynamism in art, we aim to offer new insights into the study of visual culture in the growing field of digital humanities.

## 2. Method

This section describes the whole process that we use to extract and compare the edges of the digitized paintings. It is divided into three parts: the preprocessing steps, the edge computation itself, and the computation and comparison of dynamism-related metrics. Most of these steps are relatively standard image processing techniques. The experienced reader might thus only skim through the section, while the interested reader will find the necessary information to understand our method and can find more details in a seminal textbook such as [59].

### 2.1. Preprocessing

In order to harmonize the analysis as much as possible across the paintings, we perform three preprocessing steps to remove potential artifacts from interfering with the rest of the process.

**(a) Image rescaling.** Digitized paintings are saved numerically as regular 3-channel RGB images with pixel values ranging from 0 to 255 and whose dimensions may vary from one digitization tool to another, be it a specialized hardware or a simple camera. Given that we will use a kernel method (with Sobel filters, as described hereafter) to compute edges, the resolution of the images might unnecessarily influence the analysis, as two versions of the same image but at different resolutions will respond differently to the filters. Therefore, we first resize all the images while keeping their original aspect ratio, such that they roughly have the same number of pixels as a square n×n image. This is performed by multiplying the original height *h* and width *w* by a constant factor as follows:

(1) $$h^{\prime }=\frac{hn}{\sqrt{hw}}\;\mathrm{and}\;w^{\prime }=\frac{wn}{\sqrt{hw}}$$

and rounding these values to the nearest integer. In this work, we choose n=512, which offers a good compromise: this resolution is large enough to see all the relevant details of the paintings, small enough to avoid memory allocation errors and long computation times, and close enough to the original dimensions of many digitized paintings that can be found publicly. Hence, most images do not suffer a drastic resizing, avoiding the loss of too much quality for high-resolution images and avoiding too much interpolation for low-resolution images.

**(b) Conversion to grayscale.** Edge detection is typically performed on grayscale images because it simplifies the process, improves computational efficiency, strengthens edge detection accuracy, and reduces noise interference. Grayscale images have a single channel representing intensity, making it easier to analyze and detect edges compared to color images with multiple channels. For that purpose, while various conversions exist (see, e.g., [60]), we use the commonly used formula that computes the monochrome luminance *y* of a color (r,g,b) as the following weighted sum of the channels:

(2) y=0.299∗r+0.587∗g+0.114∗b

which is supposed to be a close approximation of how humans perceive the relative brightness of red, green, and blue light [61]. This gives a single-channel grayscale image.

**(c) Pixel values rescaling.** Finally, the pixel values of the grayscale image are divided by 255 such that they range from 0 to 1, for the sake of easier interpretation of our process. This common practice is often used in image processing, e.g., when training neural networks, to keep the weights, logits, and gradients within the network within a reasonable range and facilitate the learning process.

### 2.2. Computing Edges and Corrective Normalization

This section presents how Sobel filters are used to compute directional gradients, how these are further processed to compute edge direction and magnitude, and how this magnitude is corrected depending on the computed direction such that the same maximal value can be reached regardless of the direction of the edge at hand.

**(a) Compute edges with Sobel filters.** We use Sobel filters to compute the edges (edges are defined as significant changes in pixel intensity, typically marking the boundary between two distinct regions in an image. A single pixel that is part of an edge, representing a local, discrete unit of that edge, is called an edgel. This latter terminology is less used, which makes us use the term “edge” wherever “edgel” might have been technically more appropriate. We believe that the context of the occurrence of this word makes it sufficiently clear which concept is actually at stake.) in an image [42,62]. As this is a relatively common way of proceeding, we only briefly summarize the computations hereafter. First, the grayscale image A is convolved with the Sobel filters to obtain the horizontal and vertical gradient images $G_{x}$ and $G_{y}$ as:

(3) $$G_{x}=\left[\begin{array}{c} \begin{array}{ccc} -1 & 0 & 1\\ -2 & 0 & 2\\ -1 & 0 & 1 \end{array} \end{array}\right]*A,\;\;G_{y}=\left[\begin{array}{c} \begin{array}{ccc} -1 & -2 & -1\\ 0 & 0 & 0\\ 1 & 2 & 1 \end{array} \end{array}\right]*A.$$

Then, the gradient magnitude G and direction Θ images are computed as

(4) $$G=\sqrt{G_{x}^{2}+G_{y}^{2}},\;\Theta =\arctan2\left(G_{y},G_{x}\right),$$

where arctan2(y,x) is equivalent to the argument of the complex number x+iy. Let us note that other classic methods, such as Canny edge detection [63], or more recent and sophisticated methods, e.g., based on wavelets [64], shearlets [65,66], or symmetric molecules [67], might be used as well. Our early experiments with such methods did not reveal significant qualitative improvements, which motivated the choice of Sobel filters for the sake of simplicity, control, and speed.

**(b) Magnitude normalizing factors.** To better compare and aggregate edge magnitudes per direction, we rescale the computed magnitudes by the maximum magnitude achievable per direction. More precisely, a perfectly vertical edge between a black (0-valued) and a white (1-valued) surface responds to the filters with a horizontal gradient of 4 and a vertical gradient of 0, which gives a gradient magnitude of 4 and is the maximum magnitude achievable for the gradient direction of 0 radians. Hence, we renormalize the gradient magnitude of a pixel with gradient direction 0 and magnitude 4 to 1 (that is, 100% of the maximal value achievable for that gradient direction). Similarly, a pixel with gradient direction 0 and magnitude 2 has its magnitude renormalized to 0.5. For this direction, the scaling factor is 4. However, a perfectly ascending diagonal edge of 45° between a black and a white surface responds to the filters with a horizontal gradient of 3 and a vertical gradient of 3, which gives a gradient magnitude of $3\sqrt{2}$ (the maximum achievable for that direction). This shows that the rescaling factor should be adapted to the gradient direction to reflect which fraction of the maximal value is achieved for a given direction and to allow a better comparability across the directions.

We show in Appendix A that, for a direction θ∈[0,arctan2(0.5)], the maximal magnitude is given by 4/cos(θ), and for θ∈[arctan2(0.5),π/4], it is given by 6/(cos(θ)+sin(θ)). The maximal magnitude for the other directions can be obtained by symmetries in the problem.

**(c) Per pixel edge magnitude and direction.** All in all, if a pixel has a gradient direction θ (in radians) and magnitude *g* following Equation (4), then we compute

(5) $$\theta^{\prime }=\theta \;\mathrm{mod}\;\frac{\pi }{2}\;\mathrm{and}\;\theta^{\prime \prime }=\left\{\begin{array}{cc} \theta^{\prime } & \mathrm{if}\;\theta^{\prime }<\frac{\pi }{4}\\ \frac{\pi }{2}-\theta^{\prime } & \mathrm{if}\;\theta^{\prime }\geq \frac{\pi }{4} \end{array}\right.$$

and

(6) $$\mathrm{edge}\text{\_}\mathrm{magnitude}=\left\{\begin{array}{cc} \frac{g\cos(\theta^{\prime \prime })}{4} & \mathrm{if}\;\theta^{\prime \prime }<\arctan\left(0.5\right)\\ \frac{g(\cos\left(\theta^{\prime \prime }\right)+\sin\left(\theta^{\prime \prime }\right))}{6} & \mathrm{if}\;\theta^{\prime \prime }\geq \arctan\left(0.5\right). \end{array}\right.$$

This gives the corrected edge magnitude that we will use in practice.

The edge direction is perpendicular to the gradient direction. In addition, the gradient direction differs by 180° depending on whether the contrast goes from dark to light or vice versa, which is a distinction that we do not need (we only focus on edges as such). Therefore, we limit the allowed edge direction to the range −90° to 90°, where directions at these two extreme values both depict verticality. Mathematically, we compute the edge direction that passes through a pixel of gradient direction θ as

(7) $$\mathrm{edge}\text{\_}\mathrm{direction}=\frac{\pi }{2}-\left(\theta \;\mathrm{mod}\;\pi \right).$$

Let us note that this is where we end our edge detection, i.e., we obtain per pixel values for edge magnitude and direction. We do not try to connect pixels together to form lines or anything alike, because this is highly non trivial, it loses the information of the local direction of the edge (e.g., if the edge is curved), and, in artworks, some subtle traits might not be connected as edges while they are intentional (e.g., strokes in impressionist paintings). Thus, we believe that the per pixel computation and representation provides the right information for our case study.

### 2.3. Visualization and Dynamism-Related Metrics

We can now visualize and compute a few metrics about the described processing of images.

**(a) Edge image.** The most “natural” visualization that we can produce is an image of the edge magnitudes, colored as a function of the edge direction. That is, for each pixel, the edge direction determines a color to use, which we choose ranging from green (−90°) to blue (−45°) to white (0°) to red (45°) to green again (90°), downscaled by the edge magnitude, such that surfaces with no edges are blacked out.

**(b) α-main edge image.** While intuitive and mathematically relevant, the edge image generally suffers from having many low-magnitude edges that appear relatively dark and are hard to distinguish with the naked eye from completely edgeless surfaces. To solve that issue, we can remove the downscaling by the edge magnitude. However, this has the effect of revealing only the edge direction for all the pixels, thus producing an extremely chaotic image, as even edgeless surfaces are colorized, usually with seemingly random colors in the defined palette. Following our experimentation, we found interesting to visualize only what we call the “α-main edges”, that is, we only color the pixels with the largest magnitude by thresholding not on individual magnitudes themselves (because a fixed threshold is hard to find consistently from one image to another) but on the proportion of the total magnitude they represent.

More precisely, let *M* denote the sum of all the computed edge amplitudes, and let α denote the desired fraction of *M* to visualize. We sort the pixels by decreasing edge magnitude, and we color (according to the scheme described above, with no downscaling) them until the sum of the magnitudes of the colored pixels is larger than αM. The remaining pixels remain black. This produces the α-main edge image. Low values of α give mostly blacked out images, while large values give quite noisy and chaotic images as mentioned above. In practice, we found that α=50% produces satisfying visualizations.

**(c) Circular histogram.** We define bins of 5° ranging from −92.5° to 92.5° and aggregate the magnitudes of the pixels whose direction falls within each bin, then we normalize the values by scaling them down by their sum to produce a classic density histogram. The values obtained per bin are assigned to the angle at the center of the bin and are interpolated linearly to produce a continuous visualization between those central angles rather than rectangular bins. We then wrap the histogram around a half-circle and symmetrize it for the sake of completeness. By doing so, we obtain a circular version of the histogram, which indicates, for a given direction, how much magnitude was computed on the image, and this is represented by pointing from the origin towards the direction in question on the histogram, making it a very intuitive and straightforward tool to understand the directions in the image.

**(d) Histogram radius.** We call the histogram radius the maximum value computed previously to build the histogram, that is, the normalized maximum total magnitude accumulated in a 5°-degree bin. A large value indicates a concentrated distribution in a particular direction, while a small value is the sign of a more uniform distribution across the directions.

**(e) Proportion of verticality, horizontality, and ascending and descending diagonality.** For a highly compressed view of how much the main directions are present relatively to each other, we compute the proportion of the histogram contained between −67.5° and −22.5° to assess descending diagonality, −22.5° and 22.5° to assess horizontality, 22.5° and 67.5° to assess ascending diagonality, and the remaining proportion assessing verticality. While the shape of the histogram carries more information, this simplified metric offers a compromise between an easy quantification and interpretation of the edges of the painting.

**(f) Average magnitude per pixel.** As an additional metric, we compute the average magnitude per pixel as the sum of all the amplitudes, divided by the number of pixels, regardless of the directions. This metric helps in quantifying how many edges are present within the image on a global level. Small values indicate that few edges are represented, while large values indicate many edges.

**(g) Distance between images.** To compare the dynamism induced by the edges and directions of two paintings, we compute the $L_{1}$ distance (i.e., Manhattan distance) between their normalized histograms (where their total area equals 1). This corresponds to the space between the two histograms if they are represented on top of each other. Our experiments with other distance metrics between distributions such as Kullback–Leibler divergence [68] and Bhattacharyya distance [69] generally gave results comparable to the $L_{1}$ distance. The Wasserstein distance [70] (i.e., Earth Mover Distance), which we adapted to account for the circularity of our histograms, has the advantage of reporting as two relatively close histograms that are slightly rotated versions of each other, contrary to the other metrics. However, such a theoretical benefit did not seem particularly useful in our experiments, and the results seemed qualitatively less appealing. Therefore, we report results obtained with the $L_{1}$ distance.

## 3. Results

**Figure 2:**
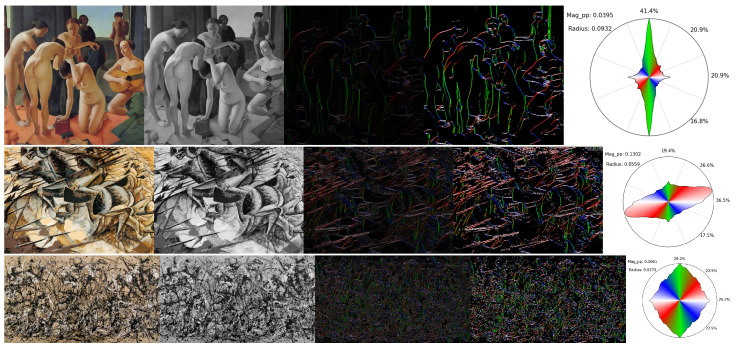
**Examples of results** of the edge detection process and visual representation with a circular histogram. First line: Felice Casorati, *Concerto*, 1924. Second line: Umberto Boccioni, *Charge of the Lancers*, 1915. Third line: Jackson Pollock, *Autumn Rhythm (Number 30)*, 1950. First column: original image. Second column: the grayscale image used for the edge detection. Third column: the edge image, where the color represents the direction of the edge and the brightness of the color represents its magnitude. Fourth column: α-main edge image (for visualization only), with α=50%, showing only the most salient edges with no downscaling on the magnitudes. Fifth column: the circular histogram aggregating the edge information and a few metrics, computed from the edge image. Mag_pp is the average magnitude per pixel.

**(a) Examples of results.** Some examples of results obtained following the process described in Section 2 are represented in Figure 2. The difference between the edge image and the α-main edge image can be clearly seen, the latter being used only for visualization purposes. For the first painting, we can observe that most salient edges are relatively vertical, given the presence of many people standing, which is correctly translated into the circular histogram, which reports over 40% of its mass in the vertical direction. For the second painting, we can see that the dominant direction is ascending diagonally, which is depicted rightfully with the red edges and the tilted shape of the circular histogram, with most of its mass pointing in the direction of roughly 20°. For the last painting, the extremely complex and dense patterns yield a much higher average magnitude per pixel (0.21 vs. 0.13 and 0.04), and these patterns spread across all directions relatively uniformly, which is represented on a more roundish histogram and roughly equivalent proportions in the four main directions (23% to 29%), with a comparatively smaller radius than the other artworks (0.04 vs. 0.06 and 0.09).

**Figure 3:**
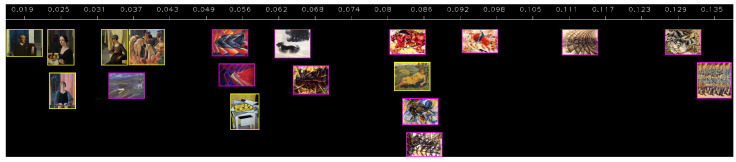
**Return to order vs. futurism comparison** in terms of average magnitude per pixel. Casorati’s paintings (yellow frames) are considered as “calm”, while images from futurism (magenta frames) are considered “dynamic”. This translates to a lower (respectively, larger) average magnitude per pixel. Artworks from futurism generally display more dynamism, represented by more salient edges and thus a larger average magnitude per pixel. Pollock’s artworks would stand far on the right, with values above 0.2.

**(b) Artistic movement vs. average magnitude per pixel.** The three results shown in Figure 2 can be seen as belonging to three different artistic movements: *Return to Order* for Casorati’s painting, *futurism* for Boccioni’s, and *abstract expressionism* through the *dripping* technique for Pollock’s work. Without being an exhaustive list of pictural movements, these intuitively drastically differ by the “amount of motion” or “dynamism” they convey on a global level. To further illustrate this, and show a first basic application of our methodology, we can represent a few paintings sorted by average magnitude per pixel, which is the metric that best captures that observation. This is represented in Figure 3, where a few paintings of Casorati are represented, along with a few paintings of the futurism period. Pollock’s artworks are not represented because their average magnitude per pixel is so large (above 0.2) that they would be on the far right of Figure 3, forcing a heavy zoom out and making it impossible to distinguish any painting. On that matter, his artworks clearly stand apart. We can also observe that most of the paintings from futurism (in magenta rectangles) have larger average magnitudes than most of Casorati’s artworks (in yellow rectangles), as intended by the futurism movement itself that wants to encapsulate dynamism in still representations. One notable exception is Casorati’s *Donna e paesaggio* (1940), which has a value within the range of most paintings of futurism. This is because that particular painting was produced with a lot of small brushes and patterns generating many edges, as they can be found in the fauvism or expressionism movements, contrary to the other, much smoother works of Casorati represented in Figure 3. Overall, this visualization accounts only for one variable, which is of course insufficient to fully characterize and distinguish major artistic movements or painters, but it shows that it can already quantify and validate some observations that could otherwise only be qualitatively assessed.

**(c) Studying the evolution of an artist: the case of Kandinsky.** Another way to use our method is to represent the evolution of the dynamism of an artist’s paintings over time. For that purpose, we choose to study the most influential paintings of Wassily Kandinsky, whose famous, prolific, and diversified work constitutes an ideal testbed for us. We collect the images referenced as most emblematic paintings of Kandinsky on Wikipedia (https://en.wikipedia.org/wiki/List_of_paintings_by_Wassily_Kandinsky, accessed on 23 May 2024), yielding a corpus of 327 images. We notice that some of the images not only show the painting itself, but also (a portion of) the frame around it. Obviously, analyzing the images with the frame gives clear horizontal and vertical edges at the intersection of the frame and the painting, which artificially boosts these directions and tends to produce more “+-shaped” circular histograms. Therefore, we manually inspect all the images and crop them to keep only the paintings. As a result, 89 images are cropped. Furthermore, to remove similar side effects that were produced by the digitization system or leftovers of the frames that we might have missed, after computing the edge magnitude for each image, we zero out the magnitudes of the 1% leftmost, rightmost, uppermost and lowermost pixels.

**Figure 4:**
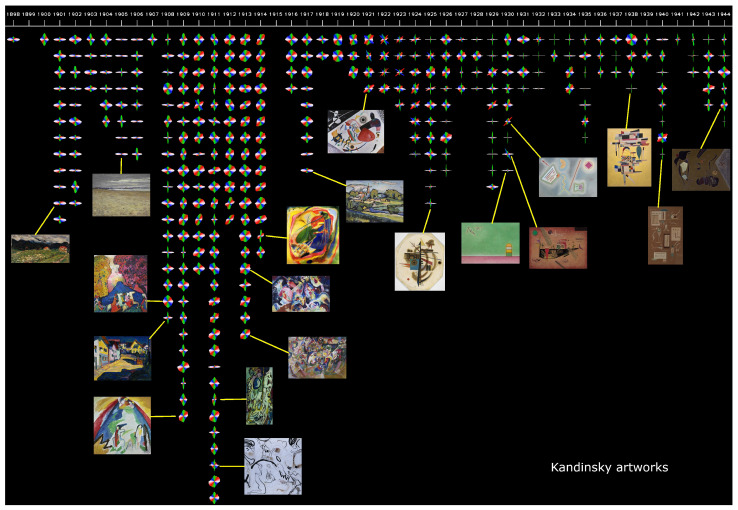
**Temporal evolution of Kandinsky** through the circular histograms of 327 of his most prominent paintings. We can observe various shifts in the overall shape of the histograms at different times, reflecting Kandinsky’s various artistic periods.

For the sake of clarity, we only represent the circular histograms along with a few artworks, sorted by date of production, in Figure 4. In this visualization, several periods of Kandinsky’s career can be distinguished through these circular histograms. We can see that, in his early career, until 1906, most of the histograms have a dominant horizontal component and usually not much verticality, as depicted in *Tunis, Coastal Landscape I* (1905). This is a sign of low dynamism and gives a sense of stability in the paintings. Then, from 1908 to 1914, we can observe that most histograms are much more roundish and uniform, such as in *Blue Mountain* (1908). As for Pollock’s art, this indicates a fuzzy dynamic, where each direction is represented equally and complex patterns are interleaved. We can also see that some paintings start to have a slightly dominant direction in the ascending diagonal, like *Draft 3 for Composition VII* (1913), which drives the eye in a slightly more dynamic way. This corresponds to Kandinsky’s Blue Rider period. We then observe, from 1916 to 1920, a small mix of the previous types of histograms already observed. This corresponds to a period where Kandinsky was in Russia and was teaching more than he was painting. From 1921 to 1925, we can observe many “x”-shaped histograms, where diagonal directions (both ascending and descending) are dominant and are characteristic of a somewhat more dynamic artwork. This particular pattern reflects Kandinsky’s increasing inclination for lines and geometric forms, such as in *Small worlds I* (1922) or *Circles in a Circle* (1923). After 1926, we observe a switch to mostly “+”-shaped histograms, indicating primarily horizontal and vertical edges. This is often the sign of a retrieved calmness and stability, sometimes to the extremes, such as in *Green Void* (1930). During that period, we sporadically note a few x-shaped histograms, uniform, or horizontally dominant ones as observed previously.

From a quantitative point of view, there is an average radius difference before 1925 and after (0.0487 vs. 0.0851), consistent with the uniform vs. +-shaped histograms observation. The average magnitude per pixel is larger before 1925 (0.0668 vs. 0.0482). These comparisons are validated as highly statistically significant by two-sample *t*-tests. We did not find any strong correlation between the radius and the average magnitude per pixel, i.e., for a given radius range, the associated magnitudes per pixel may usually vary from low to large values.

While in the case of Kandinsky, we can match known periods of his life to the dynamism (seen as the shape of the circular histograms) of his paintings, this kind of visualization might help in discovering previously unknown transition phases of many other (possibly less well-studied) artists.

**(d) A typology of histogram shapes and visual clustering of abstract paintings.** The previous section already reveals various types of histograms that can be obtained when studying a large corpus of paintings. In this section, we push the analysis further, by considering over 1000 abstract paintings and studying the distribution of the circular histograms obtained. By doing so, we establish a typology of such histograms and we gain some insight on the similarities between paintings of different artists at different times.

We consider six of the most influential abstract painters of the 20th century and download their paintings from WikiArt (https://www.wikiart.org/, accessed on 10 June 2024): Mark Rothko (163), Jackson Pollock (88), Kazimir Malevich (280), Paul Klee (193), Piet Mondrian (94), and Wassily Kandinsky (228), totaling 1046 artworks. In the case of Kandinsky, we do not reuse the Wikipedia images in order to analyze paintings from the same data source. To limit the frame effect mentioned in the previous section, and to avoid checking and cropping manually over a thousand images, we automatically zero out the magnitudes of the 5% left/right/upper/lowermost pixels. This does not prevent the frame effect when the frame is very large but still removes frame effects in many images where the frame is relatively thin or when the digitization system produced artifacts at the borders of the paintings.

**Figure 5:**
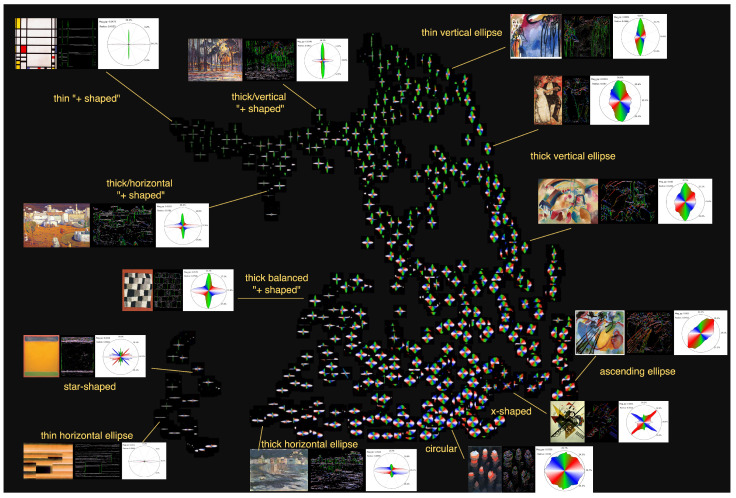
**Typology and visual clustering of histograms of abstract paintings.** We project the histograms in the 2D plane and visualize them through PixPlot. We observe several types of histograms in this dataset, usually distributed across the different painters (Kandinsky, Klee, Rothko, Malevich, Pollock, Mondrian). Interactive visualization available at http://bit.ly/4etv4Tm (accessed on 25 October 2024).

We compute the circular histograms of all the images, which can be assimilated to 36-dimensional vectors. We then project the collection of these vectors in a 2D plane with UMAP [71] by specifying the $L_{1}$ distance, as explained in Section 2, as a proximity measure to use in order to preserve as much as possible the local and global structure of the original space. Finally, the histogram images are represented where their projected vectors land in the 2D plane. We perform these operations through the PixPlot software (https://dhlab.yale.edu/projects/pixplot/, accessed on 20 July 2022), which then allows a dynamic WebGL visualization of the results. We provide access to this visualization at http://bit.ly/4etv4Tm. An overview of this visualization is shown in Figure 5. We observe various typical shapes in the histograms, usually characterized by different types of paintings. These shapes might have a dominant direction, e.g., vertical, horizontal, diagonal, yielding ellipsoid-like histograms, or a pair of dominant directions, e.g., vertical and horizontal, or ascending and descending diagonals, yielding “+”-shaped or “x”-shaped histograms, all of which might be more or less thin or thick, depending on the prominence of the non-dominant direction. When all the directions are distributed uniformly in the painting, the associated histogram has a circular shape, as no dominant direction stretches it.

Using the metadata (artist name, date) of the paintings in the WebGL application, we can further analyze the association between these shapes, the artists, and the period of time considered (we provide the temporal evolutions of these painters in Appendix B). For instance, Mondrian produced paintings with sharp “+”-shaped histograms, especially after 1918, while he produced more thin or thick horizontal ellipses beforehand. Many star-shaped histograms belong to Rothko from artworks after 1955, presumably due to subtle variations in the painting that we cannot distinguish with the naked eye on the digitized images, produced, e.g., by the technique used or the type of stroke performed, that are captured by our process and that are reflected in the analysis. He transited from a more horizontal ellipse period and before that from a more vertical or balanced “+”-shaped period to gain his distinctive style. Kandinsky produced almost all types of histograms, some more prominently in some time periods than others. Pollock’s art is generally composed of many homogeneous directions, yielding mostly circular-shaped histograms. Malevich and Klee have a more uniform production, in the sense that they produced also most of the histogram types but not necessarily at different periods of time, which can be interpreted as a sign of highly polyvalent artists, already starting from their early days.

**Figure 6:**
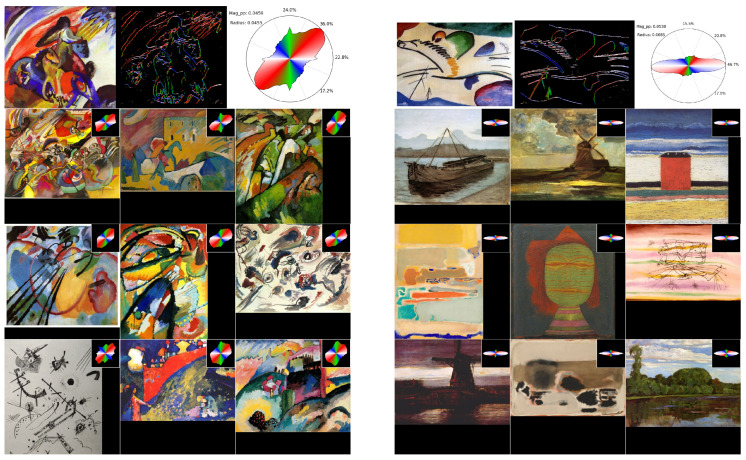
**Top 9 most similar images to a query image.** Our process allows to find similarities in paintings in terms of “dynamism”, understood as the main directions within the paintings. Left: the query image (top line) is Kandinsky’s *Improvisation 12 (Rider)* (1910). Right: the query image is Kandinsky’s *Lyrical* (1911). The retrieval corpus is the same as in the previous PixPlot visualization (6 main abstract painters).

**(e) Retrieval of most similar images in terms of histogram shape.** Let us note that the PixPlot visualization is just that: a visualization. This means that we have to keep in mind that this represents a 2D projection of a 36-dimension set of vectors. Therefore, there is necessarily a loss of information due to the information compaction, which implies that some images that appear close to each other in the 2D visualization might not be that close in the original space. Therefore, if one wants, for instance, to find the top *K* closest images to a query image in terms of circular histogram and $L_{1}$ distance, then the search should be conducted with the vectors rather than with their projection. This is what we have performed, and we show some results in Figure 6. In that case, we show, on one hand “dynamic” images with a main diagonally ascending direction and, on the other hand, more “calm” images with horizontal ellipsoids as circular histograms. This method thus allows to find similarities in paintings that are relatively difficult to observe with the naked eye or to quantify precisely and opens the door to further analyses across styles, painters, and time periods.

## 4. Limitations

We briefly discuss three limitations of the present study that would be suitable for follow-up works and improvements of our methods.

**(a) Technical limitation: frames around the paintings.** In Section 3, we either manually cropped the images to remove the frames around the paintings or we automatically removed a fraction of the computed quantities at the border of the image. However, the first case cannot scale to large databases, and the latter does not ensure that the frame is fully removed or may remove too large parts of the painting. When the frame persists, it mainly influences the vertical and horizontal amount of edges within the image, which artificially pushes it towards the category of “+”-shaped histograms, which flaws the downstream analysis. In addition, in some cases, such as Kandinsky’s *Great Resurrection* (1911), the artist paints on the frame, which thus becomes part of the artwork itself. Should it still be removed then? It may also happen that the frame produces some shadow or border effect on the digitized image, as in Kandinsky’s *Clear Air* (1901); should it be removed too? Finally, when the painter does not fill in all the space between the edges of the painting and the edge of the canvas, as in Kandinsky’s *Kochel, Lake and Herzogstand* (1902), should we consider that the edges created at the intersection are relevant? These questions will probably remain open, the answers depending on the type of analysis carried out.

**(b) Interpretative limitation: dynamism reduced to histograms.** While diagonal lines are often associated with a dynamic content, quantifying the dynamism of a painting through the sole examination of the histogram and/or the average magnitude per pixel might be an oversimplification of what dynamism is. Indeed, Pollock’s artworks contain a lot of intrinsic dynamism while having circular histograms (see, e.g., Figure 2). A slightly tilted version of Boccioni’s *Charge of the Lancers* would have a horizontal ellipsoid as a histogram, which might falsely be associated with a static content. Conversely, some of Kandinsky’s x-shaped paintings with several dominant diagonals might be considered rather static. Let us note that our analysis does not take into account the spatial distribution of the edges within the image, which might also induce different perceptions of dynamism. Other techniques are sometimes used by artists to create a sense of motion in paintings, such as the repetition of patterns or the use of curves and spirals, as in Figure 3 in paintings of the futurism movement. The use of blur, especially in photography, is also a common way to suggest that a part of the image has dynamic content without relying on specific directional traits. The contrast between the foreground and the background of the artwork can also produce the same effect, as in, e.g., M. Sokolsky’s prominent photos, where a person is in a bubble that seems to move or elevate in the foreground while being anchored in a perfectly still scenery, or in paintings with a deep perspective giving the viewer the feeling to dive into the artwork. If we even extend the notion of dynamism from motions occurring at short timescales to larger temporalities, the repeated presence of the same person at different locations of the painting has also been used to encapsulate a whole journey on a single frame, as in Sassetta’s *The Meeting of St. Anthony and St. Paul*. Subsequently, while we believe our tools provide interesting insights on the potential dynamism of paintings, it is important to keep in mind that edges, aggregated globally as performed here, constitute only one of the modalities that can be used to characterize this complex notion.

**(c) Evaluative limitation: simple approach, qualitative results.** It might be argued that the proposed approach is simplistic and reuses quite old handcrafted techniques rather than modern data-driven learning-based solutions. We believe that this is a strength more than a weakness, because this helps us produce interesting results without having the burden of collecting training data, annotating them, and training a heavy model to handle our tasks. In addition, obtaining any ground truth at all in terms of edges or dynamism in paintings is tedious, if not impossible, given the complex nature of this concept and the lack of a large-scale pool of available experts, which can hardly be solicited to examine thousands or millions of images within a reasonable timescale and budget. Moreover, a somewhat similar work [72] also achieved better results for estimating the aesthetic quality of images (a different yet interesting point of focus) through an accumulation of classic visual complexity features rather than relying on deep features extracted from neural networks. Nevertheless, we acknowledge that finding a way to provide quantitative results and, as in [72], to compare our approach with various other techniques could further validate and improve the quality of our results, although without affecting the relevance of the present experiments. Finally, an additional benefit will be on the deployment side of our method: the computational resource consumption and the speed of the processing will be by nature significantly better than with more convoluted methods. Given that part of the target audience of our work is the art history and digital humanities community, where programming skills are sometimes (but not always) limited, we seek to provide a reliable and easy-to-use application that benefits these researchers in their work. In that vein, we are confident that the results provided in this paper will convince them of the usefulness of our method and can have a significant impact in such communities.

## 5. Conclusions

Quantifying the dynamism that can be sensed in paintings is not an easy task. In this paper, we provide one way to gain insights on this problem, by computing the edges within the images and studying their distribution. By doing so, we can show some differences between artistic styles, we can study the temporal evolution of an artist across different periods of his artwork production, and we can define a typology of histogram shapes that traverse various artists, allowing the retrieval of similarly composed paintings in terms of distribution of edges. We further discuss three limitations of the present work. Our method and set of tools could certainly be improved, extended, or reused in many ways, which is why we are committed to release all code material, which will hopefully serve the digital humanities community. 

## Figures and Tables

**Figure A1:**
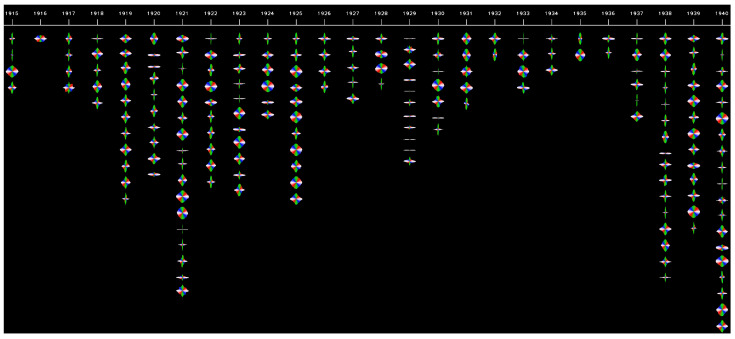
**Temporal evolution of Paul Klee** through the circular histograms of 193 of his most prominent paintings.

**Figure A2:**
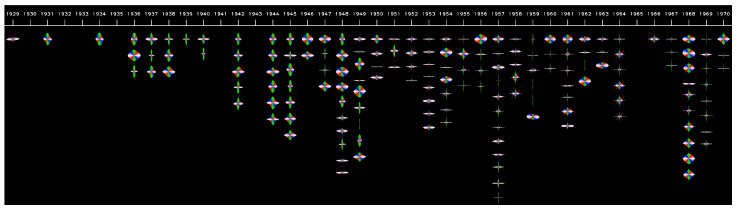
**Temporal evolution of Mark Rothko** through the circular histograms of 163 of his most prominent paintings.

**Figure A3:**
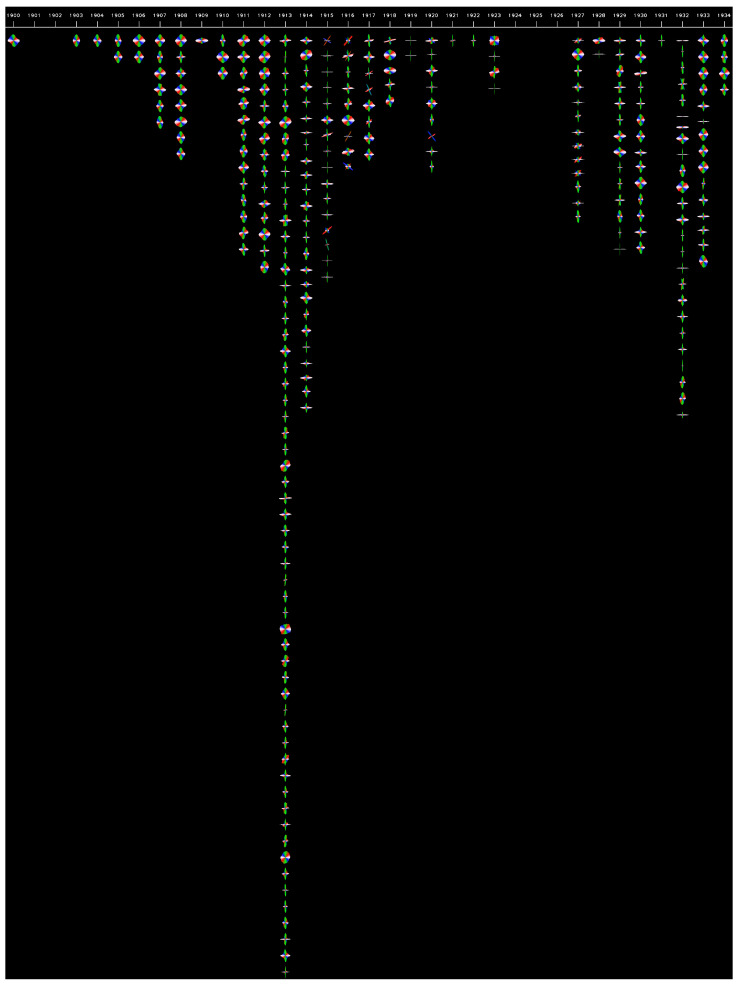
**Temporal evolution of Kazimir Malevich** through the circular histograms of 280 of his most prominent paintings.

**Figure A4:**
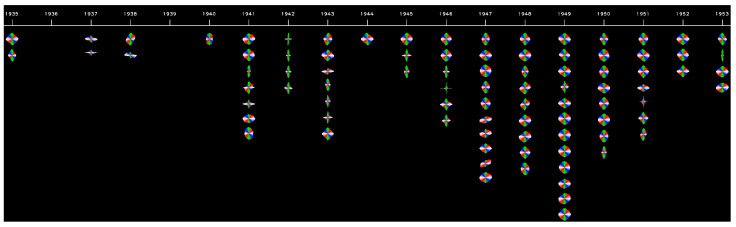
**Temporal evolution of Jackson Pollock** through the circular histograms of 88 of his most prominent paintings.

**Figure A5:**
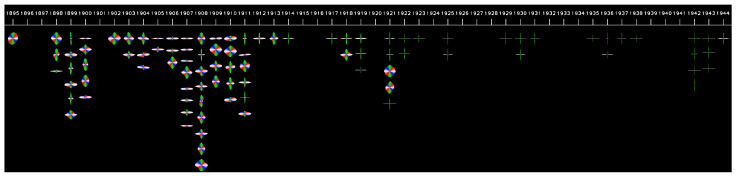
**Temporal evolution of Piet Mondrian** through the circular histograms of 94 of his most prominent paintings.

## Data Availability

All images presented in the paper are from WikiArt, Wikipedia, or generated by our algorithms. The copyright status of all the images presented in the paper is the public domain.

## Appendix A. Magnitude Normalizing Factors

We give more details about the following sentence of Section 2:

“*[...] for a direction θ∈[0,arctan2(0.5)], the maximal magnitude is given by 4/cos(θ), and for θ∈[arctan2(0.5),π/4], it is given by 6/(cos(θ)+sin(θ)). The maximal magnitude for the other directions can be obtained by symmetries in the problem.*”

Computing the theoretical maximal achievable gradient magnitude per direction can be limited to angles ranging from 0° to 45°, the rest of the proof being obtained by symmetry of the problem; i.e., angles can first be reduced by modulo 90° to the first quadrant, then to their complementary angle within that quadrant. We will use the following image patches:

$$P_{1}=\left[\begin{array}{ccc} 0 & 0 & 1\\ 0 & 0 & 1\\ 0 & x & 1 \end{array}\right],P_{2}=\left[\begin{array}{ccc} 0 & 0 & 1\\ 0 & 0 & 1\\ 0 & 2\tan(\theta ) & 1 \end{array}\right],P_{3}=\left[\begin{array}{ccc} 0 & 0 & 1\\ 0 & 0 & 1\\ x & 1 & 1 \end{array}\right],P_{4}=\left[\begin{array}{ccc} 0 & 0 & 1\\ 0 & 0 & 1\\ \frac{4\tan(\theta )-2}{1+\tan(\theta )} & 1 & 1 \end{array}\right].$$

We can first observe that the image patch $P_{1}$ with *x* varying from 0 to 1 responds maximally to the horizontal Sobel filter with a value of 4 and responds with 2x to the vertical filter, giving a direction of arctan2(x/2). This means that gradient directions from 0 to arctan2(0.5) can be achieved, and modifying any value of the patch while trying to keep the same direction can only decrease the gradient magnitude given the negative values in the filters. Conversely, if we then fix θ∈[0,arctan2(0.5)], then the image patch $P_{2}$ yields a gradient direction of θ with a magnitude of 4/cos(θ); that is, the scaling factor that we are looking for.

Similarly, $P_{3}$ with *x* varying from 0 to 1 responds to the filters with values 4−x and 2+x, giving a direction of arctan2((2−x)/(4−x)), allowing to reach all directions between arctan(0.5) and arctan(1) (corresponding to 45), with maximal magnitude. Reversing the relation by fixing an angle θ in that range gives the image patch $P_{4}$, responding to the Sobel filters with a magnitude of 6/(cos(θ)+sin(θ)), that is, the desired scaling factor.

## Appendix B. Temporal Evolution of Individual Painters

In Section 3, we provide a visualization of the temporal evolution of Kandinsky through the circular histograms. We then provide a PixPlot visualization of the distribution of 6 painters (Kandinsky, Klee, Rothko, Malevich, Pollock, Mondrian). Hereafter, we provide the temporal evolution individually for the 5 remaining painters.
